# Connections Between Numerical Algorithms for PDEs and Neural Networks

**DOI:** 10.1007/s10851-022-01106-x

**Published:** 2022-06-24

**Authors:** Tobias Alt, Karl Schrader, Matthias Augustin, Pascal Peter, Joachim Weickert

**Affiliations:** Mathematical Image Analysis Group, Faculty of Mathematics and Computer Science, Saarland University, Campus E1.7, 66041 Saarbrücken, Germany

**Keywords:** Numerical algorithms, Partial differential equations, Neural networks, Nonlinear diffusion, Stability

## Abstract

We investigate numerous structural connections between numerical algorithms for partial differential equations (PDEs) and neural architectures. Our goal is to transfer the rich set of mathematical foundations from the world of PDEs to neural networks. Besides structural insights, we provide concrete examples and experimental evaluations of the resulting architectures. Using the example of generalised nonlinear diffusion in 1D, we consider explicit schemes, acceleration strategies thereof, implicit schemes, and multigrid approaches. We connect these concepts to residual networks, recurrent neural networks, and U-net architectures. Our findings inspire a symmetric residual network design with provable stability guarantees and justify the effectiveness of skip connections in neural networks from a numerical perspective. Moreover, we present U-net architectures that implement multigrid techniques for learning efficient solutions of partial differential equation models, and motivate uncommon design choices such as trainable nonmonotone activation functions. Experimental evaluations show that the proposed architectures save half of the trainable parameters and can thus outperform standard ones with the same model complexity. Our considerations serve as a basis for explaining the success of popular neural architectures and provide a blueprint for developing new mathematically well-founded neural building blocks.

## Introduction

Partial differential equations (PDEs) have been a central component of many successful models for signal and image processing in the last three decades; for instance, the monographs [6, 16, 105] provide a good overview.

PDE-based models are compact, transparent, and benefit from a rich set of mathematical foundations. As a consequence, they offer valuable theoretical guarantees such as stability and well-posedness. This makes PDE models and their numerical solution strategies easy to control, implement, and apply to a plethora of tasks.

Convolutional neural networks (CNNs) and deep learning [39, 64, 65, 99], on the other hand, have revolutionised the field of image processing in recent years. Still, this success was mostly of empirical nature. Many modern CNN architectures do not provide solid mathematical foundations. They often suffer from undesirable side effects such as a sensitivity against adversarial attacks [40].

More recently, researchers have started to analyse the behaviour and mathematical foundations of CNNs. One strategy is to interpret trained CNNs as approximations of continuous partial or ordinary differential equations (ODEs) [19, 20, 29]. In this interpretation, the trainable parameters specify the nonlinear dynamics of an evolution equation.

This strategy can be challenging for several reasons. Firstly, finding a compact differential equation is hard, given that typical CNNs learn millions of parameters. Secondly, the reduction of a discrete CNN to a continuous differential equation involves ambiguous limit assumptions, as the same discrete model can approximate multiple evolution equations with different orders of consistency. Last but not least, this strategy is only analytic: It analyses existing networks rather than inspiring novel, well-founded building blocks.

We address these problems by pursuing the opposite direction: We translate successful concepts from the world of PDEs into neural components. This translation justifies neural architectures from a mathematical perspective and provides novel design criteria for well-founded networks. In addition, these networks are naturally more compact with less trainable parameters.

Our concepts of choice are numerical algorithms instead of continuous differential equations. Similar to untrained neural networks, numerical algorithms can be applied to a multitude of problems in a general purpose fashion. The model at hand is then specified by the differential equation, which is approximated by training the neural network. Thus, we believe that the design principles of modern neural networks realise a small but powerful set of numerical strategies at their core.

### Our Contributions

We investigate what can be learned from translating numerical methods for PDEs into their neural counterparts. This inspires novel building blocks for designing mathematically well-founded neural networks.

The present paper advances our conference publication [1], in which we translated explicit schemes, acceleration strategies [51], implicit schemes, and linear multigrid approaches [11, 12] into their neural counterparts. In this extended version, we additionally investigate networks that realise Du Fort–Frankel schemes [28] as a representative for absolutely stable schemes which are still explicit. Moreover, we extend the translation of multigrid approaches into U-nets to the nonlinear setting by considering full approximation schemes (FAS). Last but not least, we supplement our findings with an experimental evaluation of the proposed network architectures for denoising and inpainting tasks. Therein, we demonstrate the effectiveness of the architectures together with nonmonotone activation functions in practice.

As a starting point of our considerations, we consider a generalised nonlinear diffusion equation. We restrict ourselves to the 1D setting for didactic purposes only, since all important concepts can already be translated in this simple setting. We show that an explicit discretisation of this diffusion model can be connected to residual networks (ResNets) [54], which are among the most popular network architectures to date. Their skip connection can be interpreted as the result of a temporal discretisation, which allows to connect them to explicit diffusion schemes.

This connection inspires a novel ResNet architecture. It follows a symmetric structure which saves half the amount of network parameters and additionally allows to derive a theory for guaranteeing stability in the Euclidean norm. Moreover, by identifying the diffusion flux with the activation function our translation motivates the use of nonmonotone activation functions, which are atypical in the CNN world. In a series of denoising experiments, we validate the effectiveness of such activation functions. We choose the denoising problem since it is the prototypical representative of a well-posed problem, where the result depends continuously on the input data.

By considering acceleration strategies for explicit schemes and solution strategies for implicit schemes, we justify the effectiveness of skip connections in neural networks. We show that Du Fort–Frankel schemes [28], fast semi-iterative (FSI) schemes [51], and fixed point iterations for implicit schemes motivate different architectural designs which all rely on skip connections as a foundation of their efficiency.

Finally, we consider the rich class of multigrid approaches [11, 12]. We show that a nonlinear full approximation scheme (FAS) can be cast in the form of the popular U-net [87] architecture. We think that at their core, U-nets realise a multigrid strategy, and we support this claim by proposing a U-net which realises a full multigrid strategy for an inpainting task.

Our findings do not only inspire new design criteria for stable neural architectures and show that uncommon design choices can perform well in practice. They also provide structural insights into the success of popular CNN architectures from the perspective of numerical algorithms.

### Related Work

In recent years, the connection between neural networks and the world of PDEs and variational methods has become an active area of research.

CNNs are used to learn PDEs from data [68, 82, 90, 96], or to solve them efficiently [21, 30, 83]. Moreover, various model-based approaches have been augmented with trainable parameters to improve their performance [2, 5, 20, 60, 61]. Another line of research is concerned with the expressive power of networks [24, 44, 63, 79, 86, 101] and their robustness properties [23, 34, 66].

The concept of neural ordinary equations [19] as a continuous time extension of ResNets [54] has gained considerable attention. However, recent works [46, 77] suggest that these architectures suffer from a strong dependency between the model and the numerical solver. This supports our motivation to regard the numerical solver as an inherent basis of a neural architecture.

In contrast, our philosophy of translating numerical concepts into neural architectures is shared only by few works [10, 67, 69, 78, 111]. They motivate additional or modified skip connections based on numerical schemes for ODEs, such as Runge–Kutta methods or implicit Euler schemes. Our work provides additional motivations for such skip connections based on several numerical strategies for PDEs.

The stability of ResNets has been analysed in several works [17, 47, 48, 88, 93, 110]. A common result is that ResNets with a symmetric filter structure can be shown to be stable in the Euclidean norm. We motivate this result from a novel viewpoint based on diffusion processes. In contrast to previous results, this unique starting point allows us to present our stability result independently of the monotonicity of the activation function, inspiring the use of nonmonotone and trainable activation functions.

Nonmonotone activation functions are rarely found in standard CNNs, with some notable exceptions [20, 38, 76]. Recently, the so-called Swish activation [84] and modifications thereof [72, 112] have been found to empirically boost the classification performance of CNNs. While these activations are modifications of the ReLU activation which are nonmonotone around the zero position, the activations that arise from our diffusion interpretation are odd and nonmonotone. Such functions have been analysed before the advent of deep learning [25, 71], but have not found their way into current CNN architectures. Our experiments, however, suggest that these activations can be advantageous in practice.

Multigrid ideas have been combined with CNNs already in the early years of neural network research [7, 8]. Current works use inspiration from multigrid concepts to learn restriction and prolongation operators of multigrid solvers [43, 58], to couple channels for parameter reduction [31] or to boost training performance [45, 49].

However, to the best of our knowledge, the only architectures that consequently implement a trainable multigrid approach are presented by He and Xu [53] and Hartmann et al. [52]. However, both works do not draw any connections to the popular U-net architecture [87], whereas we directly link both concepts.

### Organisation of the Paper

In Sect. 2, we review nonlinear diffusion and residual networks. We connect both models in Sect. 3 and analyse the implications in terms of stability and novel activation functions. Afterwards, we motivate skip connections from different numerical algorithms in Sect. 4. We review multigrid approaches and U-nets in Sect. 5 before connecting both worlds in Sect. 6. Finally, we experimentally evaluate the proposed architectures in Sect. 7 and present a discussion and our conclusions in Sect. 8.

## Review: Diffusion and Residual Networks

In this section, we review generalised diffusion filters in 1D and residual networks as the basic models for our first translation. We restrict ourselves to the 1D setting only for didactic reasons, as already this simple setting allows to translate all necessary concepts.

### Generalised Nonlinear Diffusion

We start by considering a generalised one-dimensional diffusion PDE of arbitrary high order. It produces signals $u(x, t): (a,b) \times [0, \infty ) \rightarrow {\mathbb {R}}$ evolving over time from an initial signal *f*(*x*) on a domain $(a,b) \subset {\mathbb {R}}$ according to

1 $$\begin{aligned} \partial _t u = -{\mathcal {D}}^* \! \left( g\!\left( |{\mathcal {D}} u|^2\right) {\mathcal {D}} u\right) , \end{aligned}$$

with reflecting (homogeneous Neumann) boundary conditions. We use a general differential operator

2 $$\begin{aligned} {\mathcal {D}} = \sum _{m=0}^M \alpha _m\partial _x^m \end{aligned}$$

and its adjoint

3 $$\begin{aligned} {\mathcal {D}}^*=\sum _{m=0}^M (-1)^m \alpha _m\partial _x^m. \end{aligned}$$

The operators consist of weighted derivatives up to order *M* with weights $\alpha _m$ of arbitrary sign, yielding a PDE of order 2*M*.

Choosing e.g. $M=1$ yields the second order PDE of Perona and Malik [80], while $M=2$ leads to a one-dimensional version of the fourth order model of You and Kaveh [109].

The evolution simplifies the input signal *f* over time. This process is mainly controlled by the scalar *diffusivity* function $g(s^2)$. For example, the Perona–Malik diffusivity [80]

4 $$\begin{aligned} g(s^2) = \frac{1}{1 + \frac{s^2}{\lambda ^2}} \end{aligned}$$

preserves discontinuities which are larger than a contrast parameter $\lambda$.

The diffusion PDE (1) is the gradient flow which minimises the energy functional

5 $$\begin{aligned} E(u) = \int _a^b \Psi (|{\mathcal {D}} u|^2)\, \mathrm{d}x, \end{aligned}$$

where the penaliser $\Psi$ can be linked to the diffusivity with $g=\Psi '$ [97]. The penaliser must be increasing, but not necessarily convex. In Sect. 3.4, we show that this inspires novel activation functions. Their discretisations are stable, despite arising from a nonconvex energy.

### Residual Networks

Residual networks (ResNets) [54] belong to the most popular CNN architectures to date. Their main contribution is the introduction of so-called *skip connections* which facilitate training of very deep networks.

A residual network consists of residual blocks. A single block computes an output signal ${\varvec{u}}$ from an input ${\varvec{f}}$ as

6 $$\begin{aligned} {\varvec{u}} = \sigma _2\!\left( {\varvec{f}} + {\varvec{W}}_2 \,\sigma _1\!\left( {\varvec{W}}_1 {\varvec{f}} + {\varvec{b}}_1\right) + {\varvec{b}}_2\right) . \end{aligned}$$

One first applies an inner convolution ${\varvec{W}}_1$ with a bias vector ${\varvec{b}}_1$ to the input signal and passes the result into an inner *activation* function $\sigma _1$.

Typically, CNNs prescribe simple, monotone activation functions such as the rectified linear unit (ReLU) [73] function

7 $$\begin{aligned} \text {ReLU}(s) = \text {max}(0,s) \end{aligned}$$

which is a linear function truncated at 0.

Afterwards, an outer convolution ${\varvec{W}}_2$ with a bias vector ${\varvec{b}}_2$ is applied to the output of the activation.

The result of this convolution is added back to the original signal ${\varvec{f}}$. This *skip connection* is the crucial novelty of ResNets over feed-forward networks. It is the key to efficiently train deep networks with large amounts of layers, without suffering from the vanishing gradient problem. This phenomenon appears when backpropagation gradients approach zero for very deep networks, bringing the training process to a halt [9].

Lastly, one applies an outer activation function $\sigma _2$ to obtain the output signal ${\varvec{u}}$.

## From Diffusion to Symmetric Residual Networks

We are now in the position to show that explicit diffusion schemes realise a ResNet architecture with a symmetric filter structure. To this end, we rewrite the generalised nonlinear diffusion equation (1) with the help of the flux function

8 $$\begin{aligned} \varPhi (s) = g(s^2) \, s \end{aligned}$$

as

9 $$\begin{aligned} \partial _t u = -{\mathcal {D}}^* \varPhi \!\left( {\mathcal {D}} u\right) . \end{aligned}$$

Now we discretise this equation by means of a standard finite difference scheme. To obtain discrete signals ${\varvec{u}}, {\varvec{f}}$, we sample the continuous signals *u*, *f* with distance *h*. We discretise the temporal derivative by a forward difference with time step size $\tau$. The spatial derivative operator ${\mathcal {D}}$ is implemented by a convolution matrix ${\varvec{K}}$. Consequently, the adjoint operator ${\mathcal {D}}^*$ is realised by a transposed convolution matrix ${\varvec{K}}^\top$. The matrix transposition corresponds mirroring the corresponding discrete convolution kernel.

This yields the discrete evolution equation

10 $$\begin{aligned} \frac{{\varvec{u}}^{k+1} - {\varvec{u}}^k}{\tau } = - {\varvec{K}}^\top {\varvec{\varPhi }}\! \left( {\varvec{K}} {\varvec{u}}^k\right) , \end{aligned}$$

where we indicate old and new time levels by superscripts *k* and $k+1$, respectively. Solving this expression for the new signal ${\varvec{u}}^{k+1}$ yields the explicit scheme

11 $$\begin{aligned} {\varvec{u}}^{k+1} = {\varvec{u}}^k - \tau {\varvec{K}}^\top {\varvec{\varPhi }}\! \left( {\varvec{K}} {\varvec{u}}^k\right) . \end{aligned}$$

The explicit diffusion scheme (11) is closely connected to a specific residual block. To this end, we consider a residual block with input ${\varvec{u}}^k$, output ${\varvec{u}}^{k+1}$, and without bias terms, which reads

12 $$\begin{aligned} {\varvec{u}}^{k+1} = \sigma _2 \! \left( {\varvec{u}}^k + {\varvec{W}}_2 \, \sigma _1 \! \left( {\varvec{W}}_1 {\varvec{u}}^k\right) \right) . \end{aligned}$$

Now, we can directly identify the explicit scheme with a residual block as follows.

### Theorem 1

(Diffusion-inspired ResNets) An explicit step (11) of the generalised higher order diffusion scheme (1) can be expressed as a residual block (6) by

13 $$\begin{aligned} \sigma _1 = \tau \, {\varvec{\varPhi }}, \quad \sigma _2 = Id , \quad {\varvec{W}}_1 = {\varvec{K}}, \quad {\varvec{W}}_2 = -{\varvec{K}}^\top , \end{aligned}$$

with the bias vectors ${\varvec{b}}_1$, ${\varvec{b}}_2$ set to ${\varvec{0}}$.

**Fig. 1:**
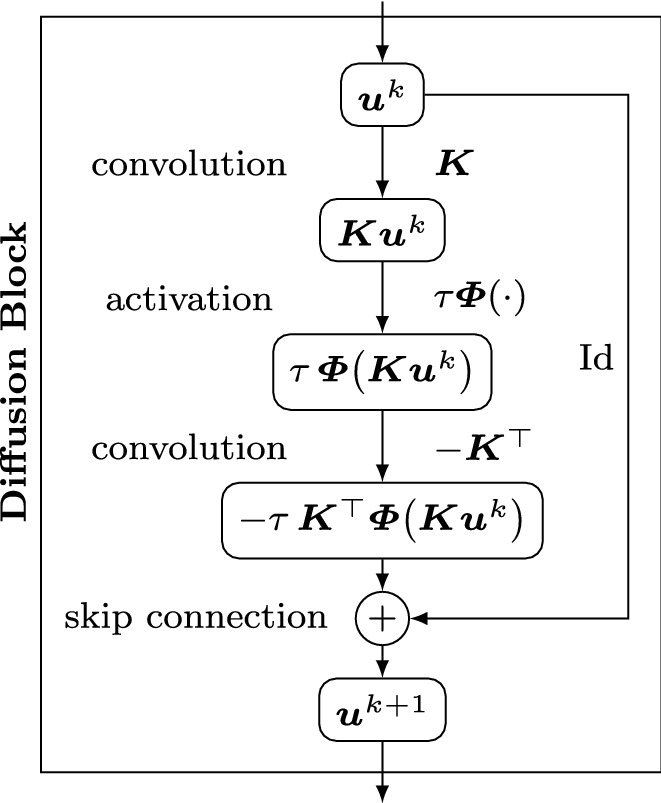
Diffusion block for an explicit diffusion step (11) with flux function ${\varvec{\varPhi }}$, time step size $\tau$, and a discrete derivative operator ${\varvec{K}}$

We call a ResNet block of this form a *diffusion block*. Figure 1 visualises such a block in the form of a graph. Nodes contain the current state of the signal, while edges describe the operations to proceed from one node to the next.

The connection between explicit diffusion schemes and ResNets yields three key structural insights:

1. The rescaled flux function $\tau \,{\varvec{\varPhi }}$ serves as the sole activation function $\sigma _1$. This motivates us to investigate popular diffusion flux functions in Sect. 3.4. They have not been considered as CNN activations so far.
2. The skip connection naturally arises from the discretisation of the temporal derivative. This is one numerical justification for skip connections in neural networks. We investigate several other motivations of skip connections in Sect. 4.
3. Lastly, the filters exhibit a negated symmetric filter structure ${\varvec{W}}_2=-{\varvec{W}}_1^\top$. This is a natural consequence of the gradient flow structure of the diffusion process, and leads to provable stability guarantees for ResNets with such a filter structure, as we show in the following.

### Stability for Symmetric ResNets

The structural connection between explicit schemes and ResNets allows us to transfer classical results for stability [26] and well-posedness [105] of diffusion evolutions to a specific residual network architecture.

To this end, we consider ResNets which chain diffusion blocks. Since we show that a key to stability of these networks is the symmetric filter structure, we refer to these architectures as *symmetric residual networks (SymResNets)*, following [93, 110].

For these networks, we prove Euclidean stability and well-posedness. Euclidean stability guarantees that the Euclidean norm of the signal is nonincreasing in each iteration, i.e. $||{\varvec{u}}^{k+1}||_2 \le ||{\varvec{u}}^k||_2$. Well-posedness ensures that the network output is a continuous function of the input data.

#### Theorem 2

(Euclidean Stability of Symmetric

Residual Networks) Consider a symmetric residual network chaining any number of diffusion blocks (11) with convolutions represented by a convolution matrix ${\varvec{K}}$ and activation function $\tau {\varvec{\varPhi }}$. Moreover, assume that the activation function can be expressed as a diffusion flux function $\varPhi (s) = g(s^2) \, s$ and has a finite Lipschitz constant *L*. Then the symmetric residual network is well-posed and stable in the Euclidean norm if

14 $$\begin{aligned} \tau \le \frac{2}{L ||{\varvec{K}}||_2^2}. \end{aligned}$$

Here, $||\cdot ||_2$ denotes the spectral norm which is induced by the Euclidean norm.

#### Proof

The activation function $\sigma (s)$ can be expressed in terms of a diffusivity function by

15 $$\begin{aligned} \sigma (s)=\tau \varPhi (s) = \tau g(s^2) \, s. \end{aligned}$$

Thus, its application is equivalent to a rescaling with a diagonal matrix ${\varvec{G}}({\varvec{u}}^k)$ with $g(({\varvec{K}} {\varvec{u}}^k)^2_i)$ as *i*-th diagonal element. Therefore, we can write (11) as

16 $$\begin{aligned} {\varvec{u}}^{k+1} = \left( {\varvec{I}} - \tau {\varvec{K}}^\top {\varvec{G}}({\varvec{u}}^k) {\varvec{K}} \right) {\varvec{u}}^k. \end{aligned}$$

At this point, well-posedness follows directly from the continuity of the operator ${\varvec{I}} - \tau {\varvec{K}}^\top {\varvec{G}}({\varvec{u}}^k) {\varvec{K}}$, as the diffusivity *g* is assumed to be smooth [105].

We now show that the time step size restriction (14) guarantees that the eigenvalues of the operator always lie in the interval $\left[ -1, 1\right]$. Then, the explicit step (11) constitutes a contraction mapping which in turn guarantees Euclidean stability.

As the spectral norm is sub-multiplicative, we can estimate the eigenvalues of ${\varvec{K}}^\top {\varvec{G}}({\varvec{u}}^k) {\varvec{K}}$ for each matrix separately. Since *g* is nonnegative, the diagonal matrix ${\varvec{G}}$ is positive semidefinite. The maximal eigenvalue of ${\varvec{G}}$ is then given by the supremum of *g*, which can be bounded by the Lipschitz constant *L* of $\varPhi$:

17 $$\begin{aligned} L =\underset{s}{\text {sup}} \left| \varPhi '(s)\right|= & {} \underset{s}{\text {sup}} \left| g(s^2) + 2 s^2 g^\prime (s^2)\right| \nonumber \\&\ge \underset{s}{\text {sup}} \left| g(s^2)\right| . \end{aligned}$$

Consequently, the eigenvalues of ${\varvec{K}}^\top {\varvec{G}}({\varvec{u}}^k) {\varvec{K}}$ lie in the interval $\left[ 0, \tau L ||{\varvec{K}}||_2^2\right]$.

Then, the operator ${\varvec{I}} - \tau {\varvec{K}}^\top {\varvec{G}}({\varvec{u}}^k) {\varvec{K}}$ has eigenvalues in $\left[ 1 - \tau L ||{\varvec{K}}||_2^2, 1\right]$, and the condition

18 $$\begin{aligned} 1 - \tau L ||{\varvec{K}}||_2^2 \ge -1 \end{aligned}$$

leads to the bound (14). $\square$

Similar results have been obtained recently in [88, 93, 110], albeit with alternative justifications. In Sect. 3.4, we show that our unique diffusion interpretation additionally suggests novel design concepts for CNNs such as nonmonotone activation functions.

### How General is Our Stability Result?

While our focus on explicit diffusion schemes appears restrictive at first glance, our stability result is more general.

The fact that we use discrete differential operators as convolutions is no restriction, since any convolution matrix can be expressed as a weighted combination of discrete differential operators. Moreover, our proof does not even require a convolutional matrix structure.
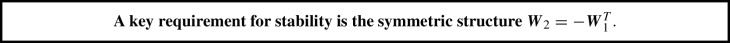


The symmetric convolution structure is an important structural difference to the original ResNet formulation [54]. It does not only yield a stable network, but also allows to reduce the amount of trainable parameters by 50%, since inner convolution and outer convolution share their weights.

Moreover, the requirement of using a flux function as an activation function can be relaxed. As we have shown, one only requires the diagonal matrix ${\varvec{G}}$ to be positive semidefinite. While this is naturally fulfilled for a diffusion flux function, other activations also adhere to this constraint. For example, the ReLU function multiplies positive arguments with 1 and negative ones with 0, yielding a binary positive semidefinite matrix ${\varvec{G}}$. Thus, using the ReLU instead of a diffusion flux does not affect stability. This shows that diffusion algorithms inspire general, sufficient design criteria for stable networks.

In particular, we do not require any assumptions on the monotonicity of the activation function, in contrast to the results of Ruthotto and Haber [93]. This motivates us to investigate typical diffusivities and their flux functions in Sect. 3.4.

### Enforcing Stability in Practice

While the stability criterion (14) can be computed on the fly already during the training process of the network, evaluating the spectral radius of the operator ${\varvec{K}}$ is costly. To this end, we suggest a simple rescaling to turn the stability bound (14) into an a priori criterion.

For a symmetric residual network with a single channel, one can directly use Gershgorin’s circle theorem [35] to bound the maximum absolute eigenvalue of ${\varvec{K}}$. More precisely, the eigenvalues of ${\varvec{K}}$ lie in the union of circles around the diagonal entries $k_{ii}$ with radii $r_i = \sum _{j\ne i} |k_{ij}|$ corresponding to the absolute sums of the off-diagonal values. Thus, the maximal absolute eigenvalue of ${\varvec{K}}$ is bounded by the largest absolute row sum of ${\varvec{K}}$. If we simply rescale both inner and outer convolutions by this sum, we can guarantee $||{\varvec{K}}||_2^2 \le 1$. Then the stability condition (14) transforms into $\tau \le \frac{2}{L}$. Since the Lipschitz constant *L* of the activation is known a priori, this simple rescaling allows to constrain the time step size to a fixed value, while not affecting the expressive power of the network.

However, most networks in practice are not concerned with only a single channel. To this end, we extend our stability result to symmetric ResNets with multiple channels.

For a diffusion block operating on a signal with *C* channels, the matrix ${\varvec{K}}$ is a $C \times C$ block convolution matrix. As long as the transposed structure is realised, this is not problematic for the stability proof.

An extension of Gershgorin’s circle theorem to block matrices [102] states that the eigenvalues of ${\varvec{K}}$ lie in the union of circles which are centred around the eigenvalues of the diagonal blocks. The radii of the circles are given by the sum of the spectral norms of the off-diagonal blocks. If we rescale each block matrix as in the single channel case, we simply need to additionally divide the operator ${\varvec{K}}$ by $\sqrt{C}$ to ensure that $||{\varvec{K}}||_2^2 \le 1$. With this, we obtain the same a priori criterion as in the single channel case.

This strategy constitutes an instance of the popular weight normalisation technique [95], and related spectral normalisations have shown to be successful for improving the performance and convergence speed of the training process [15, 22, 42, 113].

### Nonmonotone Activation Functions

**Fig. 2:**
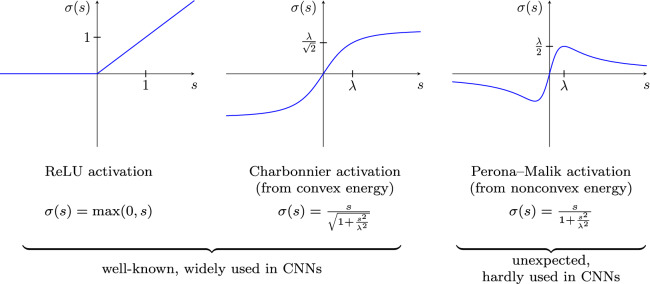
Visualisation of three activation functions. The ReLU (left) is a standard activation function. The Charbonnier and Perona–Malik activations represent two representatives of diffusion-inspired activation functions. Both are dependent on a contrast parameter $\lambda$. The Charbonnier activation is monotone, as it arises from a convex energy. On the contrary, the Perona–Malik activation is nonmonotone, and its associated energy is nonconvex. In this visualisation, we set $\tau =1$ for the sake of simplicity

Our connection between diffusivity $g(s^2)$ and activation function $\sigma (s) = \tau \, \varPhi (s)$ with the diffusion flux $\varPhi (s)$ refreshes an old idea of neural network design [25, 71].

In Fig. 2, we present three activation functions: The ReLU activation [73], along with two activations resulting from popular diffusivities. These activations are *odd* functions, which is natural in the diffusion case, where the argument of the flux function consists of signal derivatives. It reflects the invariance axiom that signal negation and filtering are commutative.

The Charbonnier diffusivity [18], which stems from a convex energy and can be seen as a rescaled regularised total variation diffusivity [4, 89], yields a monotone activation function. Similarly shaped activation functions such as the hyperbolic tangent have been used in early neural networks, before being superseded by ReLU activations.

The rational Perona–Malik diffusivity (4) [80], however, results in a nonmonotone activation function. The associated energy functional is nonconvex. Nevertheless, discretisations of diffusion processes using nonmonotone flux functions can be shown to be well-posed, despite acting contrast-enhancing [106]. For more activation functions inspired by diffusivities, we refer to [3].

The concept of nonmonotone activation functions is unusual in the CNN world. Although there have been a few early proposals in the neural network literature arguing in favour of nonmonotone activations [25, 71], they are rarely used in modern CNNs. In practice, they often fix the activation to simple, monotone functions such as the rectified linear unit (ReLU). From a PDE perspective, this appears restrictive. The diffusion interpretation suggests that activation functions should be learned in the same manner as convolution weights and biases.

In practice, this hardly happens apart from a few notable exceptions such as [20, 38, 76]. Recently, activation functions which are slightly nonmonotone variants of the ReLU proved successful for image classification tasks [72, 84, 112]. As nonmonotone flux functions outperform monotone ones, e.g. for diffusion-based denoising [80], it appears promising to incorporate them into CNNs.

Our translation of explicit schemes is an example of a simple, direct correspondence which in turn allows for multiple novel insights. In the following, we explore variants of explicit schemes as well as implicit schemes which inspire changes to the skip connections of the symmetric ResNets, leading to more efficient architectures.

## The Value of Skip Connections

So far, we have seen that the temporal discretisation of an explicit scheme naturally leads to skip connections. This, however, is just one of the many justifications for their use. Since their proposal, skip connections [54] have been adapted into numerically inspired networks in many different forms, see e.g. [56, 67, 69, 78, 111].

This motivates us to explore several other numerical algorithms which justify several types of skip connections from a numerical perspective. We explore unconditionally stable schemes, acceleration strategies for explicit schemes, and fixed point iterations for implicit schemes.

### Du Fort–Frankel Schemes

While the classical explicit scheme (11) is only conditionally stable, there exist absolutely stable schemes which are still explicit. These schemes are not that popular in practice since they trade unconditional stability for conditional consistency. However, we will see that this is not problematic from the perspective of learning.

Du Fort and Frankel [28] propose to change the temporal discretisation of the explicit scheme (11) to a central difference and introduce a stabilisation term on the right hand side, corresponding to an approximation of $\partial _{tt} u$. A Du Fort–Frankel scheme for the generalised diffusion (1) evolution can be written as

19 $$\begin{aligned} \frac{{\varvec{u}}^{k+1} - {\varvec{u}}^{k-1}}{2 \tau } =&- {\varvec{K}}^\top {\varvec{\varPhi }}\!\left( {\varvec{K}} {\varvec{u}}^k\right) \nonumber \\&- \alpha \left( {\varvec{u}}^{k+1} - 2 {\varvec{u}}^k + {\varvec{u}}^{k-1}\right) , \end{aligned}$$

where a positive constant $\alpha$ controls the influence of the stabilisation term.

Solving this scheme for ${\varvec{u}}^{k+1}$ yields

20 $$\begin{aligned} {\varvec{u}}^{k+1}&= \frac{4\tau \alpha }{1 +2\tau \alpha }\left( {\varvec{u}}^k- \frac{1}{2\alpha }{\varvec{K}}^\top {\varvec{\varPhi }}\!\left( {\varvec{K}} {\varvec{u}}^k\right) \right) \nonumber \\&\qquad \qquad \qquad \qquad \quad + \frac{1 - 2\tau \alpha }{1 + 2\tau \alpha } {\varvec{u}}^{k-1}. \end{aligned}$$

For $\tau \alpha = \frac{1}{2}$, one obtains the explicit scheme (11).

The scheme involves the signals ${\varvec{u}}^k$ and ${\varvec{u}}^{k-1}$ at the current and the previous time level. The first term is nothing else than a rescaled diffusion block, where $\frac{1}{2 \alpha }$ takes the role of the original time step size. Since the scalar factors $\frac{4\tau \alpha }{1 +2\tau \alpha }$ and $\frac{1 - 2\tau \alpha }{1 + 2\tau \alpha }$ add up to 1, this is simply an extrapolation of the result of an explicit step based on the signal at time level $k-1$.

If $\alpha$ is large enough, this scheme is unconditionally stable. Thus, one does not need to obey any stability condition, in contrast to the explicit case. Whereas classical proofs such as [41] consider only the linear case and typically work in the Fourier space, we are not aware of any proofs for the stability of nonlinear Du Fort–Frankel schemes. To this end, we prove stability of the nonlinear case in Appendix 1.

However, this scheme is not unconditionally consistent. If the time step size $\tau$ is too large, the scheme (20) approximates a different PDE [28], namely a nonlinear variant of the telegrapher’s equation. Such PDEs have also been used in image processing, see e.g. [85].

In the trainable setting, the conditional consistency is not an issue, but can even present a chance. It allows the network to learn a more suitable PDE for the problem at hand. In our experiments, we show that indeed, the unconditional stability of the Du Fort–Frankel scheme can help to achieve better results when only few residual blocks are available.

This scheme can be realised with a small change in the original diffusion block from Fig. 1 by adding an additional skip connection. The two skip connections are weighted by $\frac{4\tau \alpha }{1 +2\tau \alpha }$ and $\frac{1 - 2\tau \alpha }{1 + 2\tau \alpha }$, respectively.

### Fast Semi-Iterative Schemes

Another numerical scheme which also leads to the same concept from a different motivation is based on acceleration strategies for explicit schemes. Hafner et al. [51] introduced fast semi-iterative (FSI) schemes to accelerate explicit schemes for diffusion processes.

In a similar manner as the Du Fort–Frankel schemes, FSI extrapolates the diffusion result at a fractional time step $k+\frac{\ell }{L}$ with the previous fractional time step $k+\frac{\ell -1}{L}$ and a weight $\alpha _\ell$. For the explicit diffusion scheme (11), an FSI acceleration with cycle length *L* reads

21 $$\begin{aligned} {\varvec{u}}^{k+\frac{\ell +1}{L}}&= \alpha _\ell \left( {\varvec{u}}^{k+\frac{\ell }{L}} - \tau {\varvec{K}}^\top {\varvec{\varPhi }}\!\left( {\varvec{K}} {\varvec{u}}^{k+\frac{\ell }{L}}\right) \right) \nonumber \\&\qquad \qquad \qquad \quad + \left( 1-\alpha _\ell \right) {\varvec{u}}^{k+\frac{\ell -1}{L}} \end{aligned}$$

with fractional time steps $\ell =0,\dots ,L\!-\!1$ and extrapolation weights $\alpha _\ell {:=} (4\ell +2)/(2\ell +3)$. One formally initialises with ${\varvec{u}}^{k-\frac{1}{L}} {:=} {\varvec{u}}^{k}$.

The crucial difference to Du Fort–Frankel schemes is that FSI schemes use time-varying extrapolation coefficients instead of fixed ones. These coefficients are motivated by a box filter factorisation and allow a cycle to realise a super time step of size $\frac{L(L+1)}{3}\tau$. Thus, with one cycle involving *L* steps, one reaches a super step size of ${\mathcal {O}}(L^2)$ rather than ${\mathcal {O}}(L)$. This explains its remarkable efficiency [51].

**Fig. 3:**
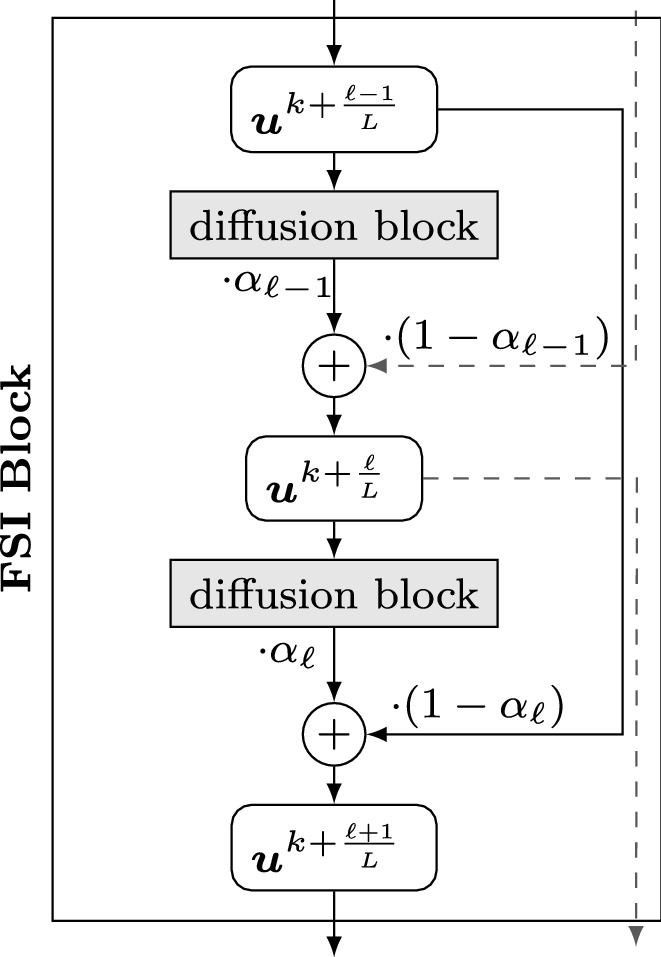
FSI block realising the acceleration of an explicit diffusion step (21) with time-varying extrapolation parameters $\alpha _\ell$. A similar architecture with differently weighted skip connections arises for a Du Fort–Frankel scheme (20)

Even though Du Fort–Frankel and FSI schemes have fundamentally different motivations, they lead to the same architectural changes, where additional weighted skip connections realise acceleration strategies. This is in line with observations in the CNN literature, see e.g. [69, 78]. We visualise this concept at the example of an FSI architecture in Fig. 3.

FSI and Du Fort–Frankel schemes are just two representatives of a large class of extrapolation strategies, see e.g. [74, 81, 103]. The ongoing success of using momentum methods for training [91, 100] and constructing [69, 78, 111] neural networks warrants an extensive investigation of these strategies in both worlds.

### Implicit Schemes

So far, we have investigated variants of explicit schemes and their neural counterparts. However, implicit discretisations constitute another important solver class. We now show that such a discretisation of the generalised diffusion equation can be connected to a recurrent neural network (RNN). RNNs are classical neural network architectures, see e.g. [55]. At the same time, this translation inspires yet another way of leveraging skip connections.

A fully implicit discretisation of the diffusion equation (1) is given by

22 $$\begin{aligned} {\varvec{u}}^{k+1} = {\varvec{u}}^k - \tau {\varvec{K}}^\top {\varvec{\varPhi }}\!\left( {\varvec{K}} {\varvec{u}}^{k+1}\right) . \end{aligned}$$

The crucial difference as opposed to the explicit scheme lies in using the new signal ${\varvec{u}}^{k+1}$ within the flux function ${\varvec{\varPhi }}$. This yields a nonlinear system of equations, which we solve by means of a fixed point iteration with a cycle of length *L*:

23 $$\begin{aligned} {\varvec{u}}^{k+\frac{\ell +1}{L}} = {\varvec{u}}^k - \tau {\varvec{K}}^\top {\varvec{\varPhi }}\! \left( {\varvec{K}} {\varvec{u}}^{k+\frac{\ell }{L}}\right) , \end{aligned}$$

where $\ell =0,\dots ,L\!-\!1$, and where we assume that $\tau$ is sufficiently small to yield a contraction mapping.

For $L=1$, we obtain the explicit scheme (11) with its ResNet interpretation. For larger *L*, however, different skip connections arise. They connect the layer at time step *k* with all subsequent layers at steps $k+\frac{\ell }{L}$ with $\ell =0,\dots ,L\!-\!1$.

There are two possible ways of interpreting this type of connection. One option is to regard this as a consequent extension of the extrapolation idea of FSI and Du Fort–Frankel schemes. Instead of only connecting a node to its two successors, the fixed point iteration above connects a node to *L* of its successors. Similar ideas have been used in the popular DenseNet architecture [56], where each layer is connected to all subsequent ones.

Another option is to interpret the repeated connection to ${\varvec{u}}^k$ as a feedback loop, which in turn is closely connected to a recurrent neural network (RNN) architecture [55].

In their trainable nonlinear diffusion model, Chen and Pock [20] proposed a similar architecture. However, they explicitly supplement the diffusion process with an additional reaction term which results from the data term of the energy. Our feedback term is a pure numerical phenomenon of the fixed point solver.

We see that skip connections can implement a number of successful numerical concepts: forward difference approximations of the temporal derivative in explicit schemes, extrapolation steps to accelerate them, e.g. via FSI or Du Fort–Frankel schemes, and recurrent connections within fixed point solvers for implicit schemes.

## Review: Multigrid Solvers and U-nets

Although the previously investigated numerical strategies and their neural counterparts can be efficient, they work on a single scale: The signal is considered at its original resolution at all points in time. However, using different signal resolutions in a clever combination can yield even higher efficiency.

This is the core idea of the large class of multigrid approaches [11, 12, 50]. They belong to the most efficient numerical methods for PDE-related problems and have been successfully applied to various tasks such as image denoising [13], inpainting [70], video compression [62], and image sequence analysis [14].

On the CNN side, architectures that work on multiple resolutions of the signal have become very successful. In particular, the shape of the popular U-net architecture [87] suggests that there is a structural connection between multigrid and CNN concepts.

By translating multigrid solvers into a U-Net architecture, we show that this is indeed the case, which serves as a basis for explaining the remarkable success of U-nets. Since both underlying concepts are not self-explanatory, we review them in the following before connecting them in the next section.

### Multigrid Solvers for Nonlinear Systems

Multigrid methods [11, 12, 50] are designed to accelerate the convergence speed of standard numerical solvers such as the Jacobi or the Gauß–Seidel method [94]. These solvers attenuate high-frequent components of the residual error very quickly, while low-frequent error components are damped slowly. This causes a considerable drop in convergence speed after a few iterations.

Multigrid methods remedy this effect by transferring the low-frequent error to a coarser grid, transforming them into high-frequent components. This allows a coarse grid solver to attenuate them more efficiently. By correcting the fine grid approximation with coarse grid results, convergence speed can be significantly improved.

In the following, we review the so-called full approximation scheme (FAS) [11] for a nonlinear system of equations. We consider a two-grid cycle as the basic building block of more complex multigrid solvers.

We are interested in solving a nonlinear system of equations of the form

24 $$\begin{aligned} {\varvec{A}}({\varvec{x}}) = {\varvec{b}}, \end{aligned}$$

with a nonlinear operator ${\varvec{A}}$ and a right hand side vector ${\varvec{b}}$ for an unknown coefficient vector ${\varvec{x}}$.

The two-grid FAS involves two grids with different step sizes: a fine grid of size *h*, and a coarse grid of size $H~>~h$. We denote the respective grid by superscripts. The following six steps describe the two-grid FAS:

1. *Presmoothing Relaxation* A standard solver is applied to the fine grid system ${\varvec{A}}^h\!\left( {\varvec{x}}^h\right) = {\varvec{b}}^h$. Given an initialisation ${\varvec{x}}_0^h$, it produces an approximation ${\varvec{{\tilde{x}}}}^h$ to the solution with a reduced high frequency error.
2. *Restriction* In order to approximate low-frequent components of the error more efficiently, one transfers the problem to the coarse grid with the help of a restriction operator ${\varvec{R}}^{h\rightarrow H}$. One restricts both the residual ${\varvec{r}}^h = {\varvec{A}}^h\!\left( {\varvec{x}}^h\right) - {\varvec{b}}^h$ as well as the current approximation ${\varvec{{\tilde{x}}}}^h$ to the coarse grid. One obtains two parts of the right hand side for the coarse grid problem: One part ${\varvec{b}}^H = {\varvec{R}}^{h\rightarrow H} {\varvec{r}}^h$ which is used directly, and a second one which we denote by ${\varvec{y}}^H ={\varvec{R}}^{h\rightarrow H} {\varvec{{\tilde{x}}}}^h$ serving as the argument for the nonlinear operator ${\varvec{A}}^H$. The coarse grid problem then reads
  25 $$\begin{aligned} {\varvec{A}}^H\!\left( {\varvec{x}}^H\right) = {\varvec{A}}^H\!\left( {\varvec{y}}^H\right) + {\varvec{b}}^H. \end{aligned}$$
  If we express the desired solution ${\varvec{x}}^H$ in terms of the error ${\varvec{e}}^H$ by ${\varvec{x}}^H={\varvec{y}}^H + {\varvec{e}}^H$, then we see that this equation is solved for the full approximation rather than the error alone, in contrast to a linear multigrid scheme. Hence, this scheme is called the full approximation scheme. If the operator ${\varvec{A}}$ is linear, FAS reduces to a linear multigrid scheme. A standard choice for ${\varvec{R}}^{h\rightarrow H}$ is a simple averaging. However, finding suitable restriction operators is a difficult task, which motivates researchers to even learn such operators, see e.g. [43, 58].
3. *Coarse Grid Computation* Solving the coarse grid problem with a standard solver produces an error approximation ${\varvec{{\tilde{x}}}}^H$.
4. *Prolongation* The approximation on the coarse grid needs to be transferred to the fine grid again. To this end, one applies a prolongation operator ${\varvec{P}}^{H\rightarrow h}$. Since a coarse grid solution ${\varvec{{\tilde{x}}}}^H$ is a full approximation, we need to compute the approximation to the error by ${\varvec{{\tilde{x}}}}^H - {\varvec{y}}^H$. This error approximation is then transferred to the fine grid via ${\varvec{P}}^{H\rightarrow h}$. A standard choice for ${\varvec{P}}^{H\rightarrow h}$ is a nearest neighbour interpolation, but as for the restriction operator, finding a good prolongation operator is not easy.
5. *Correction* The fine grid approximation ${\varvec{{\tilde{x}}}}^h$ is corrected with the upsampled coarse grid error approximation ${\varvec{P}}^{H\rightarrow h}\!\left( {\varvec{{\tilde{x}}}}^H - {\varvec{y}}^H\right)$ to produce a new approximation
  26 $$\begin{aligned} {\varvec{{\tilde{x}}}}^h_\text {new} = {\varvec{{\tilde{x}}}}^h + {\varvec{P}}^{H\rightarrow h}\!\left( {\varvec{{\tilde{x}}}}^H - {\varvec{y}}^H\right) . \end{aligned}$$
6. *Postsmoothing Relaxation* Finally, one applies another solver on the fine grid to smooth high frequent errors which have been introduced by the correction step.

The two-grid FAS will serve as the starting point for translating multigrid concepts into the a U-net formulation, in an extension to the linear connections from our conference publication [1].

### Review: U-nets

U-nets [87] are another popular neural architecture. They process information on multiple scales by repeatedly down- and upsampling the input data, interleaved with a series of convolutional network layers. This multiscale analysis makes them well-suited for medical image analysis tasks such as segmentation [27, 87], but also for pose estimation [75] and shape generation [32].

A two-level U-net with fine grid size *h* and coarse grid size *H* has the following structure:

1. An input signal ${\varvec{f}}^h$ is fed into a series of general convolutional layers which we denote by ${\varvec{C}}^h_1\!\left( \cdot \right)$. The resulting output signal is denoted by ${\varvec{{\tilde{f}}^h}} = {\varvec{C}}^h_1\!\left( {\varvec{f}}^h\right)$. Originally, these layers are assumed to be feed-forward convolutional layers, but they can also be replaced by any other suitable layer type such as residual layers.
2. The fine grid signal ${\varvec{{\tilde{f}}^h}}$ is transferred to a coarser grid with a restriction operator ${\varvec{R}}^{h\rightarrow H}$, yielding a coarse grid signal ${\varvec{f}}^H = {\varvec{R}}^{h\rightarrow H} {\varvec{f}}$.
3. On the coarse grid, another series of convolutional layers ${\varvec{C}}^H\!\left( \cdot \right)$ is applied to the signal, yielding a modified coarse grid signal ${\varvec{{\tilde{f}}}}^H = {\varvec{C}}^H\!\left( {\varvec{f}}^H\right)$.
4. The modified coarse grid signal is upsampled with a prolongation operator ${\varvec{P}}^{H\rightarrow h}$.
5. With the help of a skip connection, the modified fine grid signal ${\varvec{{\tilde{f}}}}^h$ and the upsampled coarse grid signal ${\varvec{P}}^{H\rightarrow h}{\varvec{{\tilde{f}}}}^H$ are added together. This produces a new fine grid signal ${\varvec{{\tilde{f}}}}^h_\text {new}$. While the original U-net formulation [87] suggests to concatenate both signals, other works such as [75] simply add the signals. For our following discussion of connections between U-nets and multigrid schemes, we focus on the latter variant.
6. Lastly, another series of convolutional layers ${\varvec{C}}_2^h\!\left( \cdot \right)$ is applied to the new fine grid signal, producing the final output signal $\hat{{\varvec{f}}}^h$.

**Fig. 4:**
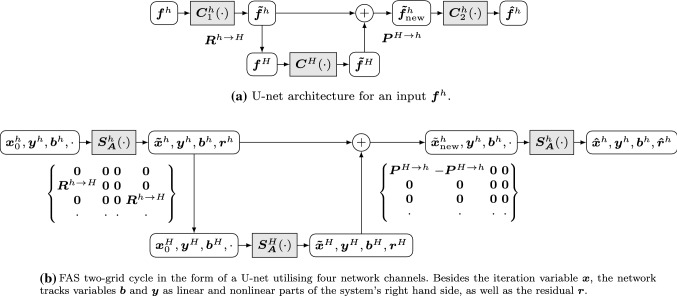
Architectures for a general U-net (**a**) and an FAS two-grid cycle (**b**)

We visualise this architecture in Fig. 4a.

## From Multigrid to U-nets

Now we show how one can express FAS in terms of a U-net architecture. For our U-net, we use multiple network channels which carry the variables required by FAS. Even though not all variables are used at each point in the network, we keep the channel number consistent for the sake of simplicity.

We track the FAS variables in dedicated channels only for didactic reasons, as a direct translation shows that a U-net architecture is sufficient for representing FAS. When practically implementing FAS, this overhead can be spared.

Firstly, let us assume that we are given suitable solvers ${\varvec{S}}_{{\varvec{A}}}^h(\cdot ), {\varvec{S}}_{{\varvec{A}}}^H(\cdot )$ for the nonlinear operators on the fine and coarse grid, respectively. To be able to use the two-grid cycle as a recursive building block, we assume that all solvers approximate solutions for nonlinear systems of the form ${\varvec{A}}({\varvec{x}}) = {\varvec{A}}({\varvec{y}}) + {\varvec{b}}$, regardless of the grid.

To this end, we always keep track of the iteration variable ${\varvec{x}}$, the nonlinear right hand side ${\varvec{y}}$, and the linear right hand side ${\varvec{b}}$. In addition, we track the residual ${\varvec{r}}$. By appropriately modifying these variables, we can ensure that the solvers always act on the desired system, despite having a common specification.

1. *Presmoothing Relaxation* The first instance of the fine grid solver obtains an initial iteration variable ${\varvec{x}}_0^h$, which can be ${\varvec{0}}$ or a more sophisticated guess. Since the first solver is supposed to solve ${\varvec{A}}^h\!\left( {\varvec{x}}^h\right) = {\varvec{b}}^h$, we simply set ${\varvec{y}}^h={\varvec{0}}$. In addition, we provide the linear right hand side ${\varvec{b}}^h$. A residual is not needed as an input. The solver produces a preliminary approximation ${\varvec{{\tilde{x}}}}^h$, and passes the right hand side components ${\varvec{y}}^h$ and ${\varvec{b}}^h$ through without changes. It also computes the residual ${\varvec{r}}^h = {\varvec{b}}^h - {\varvec{A}}^h\!\left( {\varvec{\tilde{x}}}^h\right)$ as an additional output.
2. *Restriction* As the downsampling is now explicitly concerned with four channels, the corresponding operator in our U-net is a $4\times 4$ block matrix. We apply the multigrid restriction operator only to certain channels. The coarse grid initialisation ${\varvec{x}}_0^H$ can be set to ${\varvec{0}}$, taking no information from the fine grid. The coarse, nonlinear right hand side ${\varvec{y}}^H ={\varvec{R}}^{h\rightarrow H} {\varvec{{\tilde{x}}}}^h$ is given by the downsampled fine grid approximation. The corresponding linear right hand side ${\varvec{b}}^H = {\varvec{R}}^{h\rightarrow H} {\varvec{r}}^h$ is the downsampled residual. In contrast to our linear correspondences in [1], the restriction step in FAS fits a U-net interpretation even better, since the approximation ${\varvec{{\tilde{x}}}}^h$ itself is restricted, as is the case in the U-net.
3. *Coarse Grid Computation* The coarse solver follows the same specification as the fine grid one. However, since ${\varvec{y}}^H$ is not set to ${\varvec{0}}$ at this point, the coarse solver actually solves the desired system ${\varvec{A}}^H\!\left( {\varvec{x}}^H\right) = {\varvec{A}}^H\!\left( {\varvec{y}}^H\right) + {\varvec{b}}^H$. It produces a coarse approximation ${\varvec{{\tilde{x}}}}^H$ and a residual ${\varvec{r}}^H$, while leaving the right hand side components unchanged.
4. *Prolongation* The upsampling step allows to prepare the coarse grid variables in such a way that the skip connection automatically performs the correct additions. The first row of the matrix operator ensures that we upsample the correction ${\varvec{P}}^{H\rightarrow h}{\varvec{{\tilde{x}}}}^H - {\varvec{P}}^{H\rightarrow h}{\varvec{ y}}^H$. Note that this is equivalent to the FAS formulation ${\varvec{P}}^{H\rightarrow h}\!\left( {\varvec{{\tilde{x}}}}^H - {\varvec{ y}}^H\right)$ if the prolongation operator is linear. This is no limitation, however, since for the nonlinear case we can require the solvers to directly output the difference ${\varvec{{\tilde{x}}}}^H - {\varvec{y}}^H$. The right hand side components ${\varvec{y}}^H$ and ${\varvec{b}}^H$ are not used in the upsampling, as the fine grid right hand side is supposed to be passed on. The same holds for the residual, as it is not relevant to the second fine grid solver. It is only needed in case one adds another coarser level to the cycle.
5. *Correction* In the correction step, the fine approximation ${\varvec{{\tilde{x}}}}^h$ is appropriately corrected, and the fine grid right hand side components ${\varvec{y}}^h$ and ${\varvec{b}}^h$ are forwarded.
6. *Postsmoothing Relaxation* Another instance of the fine grid solver solves the problem ${\varvec{A}}^h\!\left( {\varvec{x}}^h\right) = {\varvec{b}}^h$. The nonlinear part ${\varvec{y}}^h$ of the right hand side is still set to ${\varvec{0}}$, ensuring that the correct system is solved.

The resulting architecture is visualised in Fig. 4b. This shows that U-nets share essential structural properties with multigrid methods. In particular, employing multiple image resolutions connected through pooling and upsampling operations, as well as horizontal skip connections which realise correction steps are the keys for the success of both methods. This leads us to believe that at their core, U-nets realise a sophisticated multigrid strategy.

### V-Cycles, W-Cycles and Full Multigrid

Our connections between two-grid FAS and U-nets are the basic building block for more advanced multigrid strategies.

So-called V-cycles arise from recursively stacking the two-grid FAS. Moreover, W-cycles can be built by concatenating several V-cycles. Optimising the depth and length of these cycles can lead to vast efficiency gains over direct solution strategies.

On the CNN side, the corresponding concept of U-nets with more levels as well as concatenations thereof is successful in practice: Typical U-nets work on multiple resolutions [87], and so-called stacked hourglass models [75] arise by concatenating multiple V-cycle architectures.

A full multigrid (FMG) strategy solves a problem on multiple grids by successively concatenating V- and W-cycles, usually starting at the coarsest grid and progressing towards the finest one. In our experiments in Sect. 7.2, we will construct a trainable FMG model based on the two-grid FAS network to approximate the solution of an inpainting problem. This shows that our model reduction of the full U-net is successful in practice and inspires new design strategies for U-nets.

## Experimental Evaluations

Let us now show that our findings are also of practical relevance. Our experiments are divided into two parts. First, we evaluate the proposed symmetric ResNet architectures, along with their variations and nonmonotone activation functions for a denoising problem.

In a second experiment, we make use of our connections between multigrid and U-nets to learn an efficient solver for diffusion-based sparse inpainting, based on a trainable FAS architecture.

### Symmetric ResNets and Nonmonotone Activations

Since we motivate our network designs through numerical algorithms for a diffusion problem, we start with an elementary comparison on a denoising problem. We deliberately choose a denoising problem, since it is a prime example of a well-posed problem, for which the presented numerical schemes can be easily applied.

In a second step, we refine the simple network structures to more and more complex ones, approaching the standard neural network design. This shows the extent to which our networks can compete with off-the-shelf ResNets.

#### Experimental Setup

We compare symmetric ResNets and their Du Fort–Frankel and FSI extensions with the original ResNet architecture [54]. As activation functions we allow the ReLU [73], Charbonnier [18], and Perona–Malik [80] activation functions.

The original ResNets train two filter kernels per ResNet block, along with two bias terms. The symmetric ResNets on the other hand only train one filter kernel per block, without any bias terms. We only consider kernels of width three. For maximal transparency, we do not use any additional optimisation layers such as batch normalisation.

When using Charbonnier and Perona–Malik activations, we always train the corresponding contrast parameter $\lambda$. The Du Fort–Frankel networks also learn the extrapolation parameter $\alpha$, and the FSI networks train individual extrapolation parameters of each block.

In addition, all models train their numerical parameters such as time step size and extrapolation parameters. We restrict the time step size $\tau$ to our stability condition (14) to obtain a stable symmetric ResNet model. In the case of the Du Fort–Frankel extension we restrict the extrapolation parameter $\alpha$ to the bound in Appendix 1, thus also yielding a stable scheme. For FSI, we restrict the extrapolation parameters $\alpha _{\ell }$ to the range [0, 2]. This preserves the extrapolation character of the scheme. However, no stability theory is available in the case of learned extrapolation parameters.

**Fig. 5:**
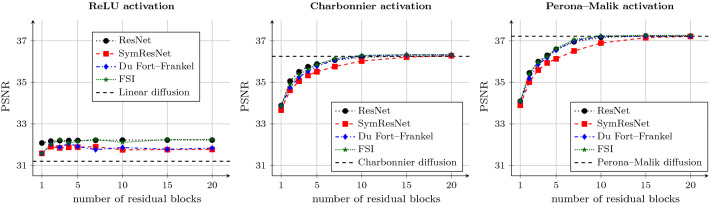
Denoising quality of network architectures with varying depth. We use a single channel, and weights between all blocks are shared. Each plot is concerned with a different activation function. Architectures with Perona–Malik activation perform best, while the ReLU activation is not suitable in this setting. Due to the tight network constraints, the architectures reproduce the performance of classical diffusion filters

We evaluate the networks on a synthetic dataset of 1D signals which are piecewise affine, with jumps between the segments. This design highlights the ability of the different approaches to preserve signal discontinuities. The signals are of length 256 and are composed of linear segments that span between $\frac{1}{10}$ and $\frac{1}{2}$ of the signal length. Their values lie within the interval [0, 255].

Finally, we add Gaussian noise of standard deviation $\sigma =10$ to the signals, without clipping out of bounds values. This yields pairs of corrupted and ground truth signals. The training dataset contains 10000 such pairs, and the test and validation datasets contain 1000 pairs each. As a measure of denoising quality, we choose the peak-signal-to-noise ratio (PSNR), where higher values indicate better denoising performance.

For a fair comparison, we train all network configurations in the same fashion. We use the Adam optimiser [59] with a learning rate of 0.001 for at most 2000 training epochs, and choose the average mean square error (MSE) over the training dataset as an optimisation objective.

The filter weights are initialised according to a uniform random distribution with a range of $[-0.1, 0.1]$. The contrast parameters $\lambda$, the time step sizes $\tau$, and the extrapolation weights $\alpha$ are initialised with fixed values of 15, 1.0, and 1.0, respectively. Out of several random initialisations, we choose the best performing one.

#### Evaluation of Model Components

We first evaluate the potential of the proposed network blocks on an individual level. To this end, we train the architectures for varying amounts of residual blocks. However, all blocks share their weights, and we also use only a single network channel.

This configuration is closest to the interpretation of explicit schemes, and it allows us to investigate the approximation qualities of the different architectures within a tightly controlled frame.

Figure 5 presents the denoising quality of the architectures in dependence of the number of residual blocks. Each plot is concerned with a different activation function.

Firstly, we compare the different network architectures. As the symmetric ResNet is guaranteeing stability and uses less than half of the parameters of the standard ResNet, it performs slightly worse. This is not surprising, since there is a natural tradeoff between performance (high approximation quality) and stability, as is well known in the field of numerical analysis. Nevertheless, when enough blocks are provided, the symmetric ResNet catches up to the standard one.

The acceleration methods of Du Fort–Frankel and FSI outperform the symmetric ResNet and yield comparable performance to the standard ResNet. The trainable extrapolation parameters help both methods to achieve better quality especially when not enough residual blocks are provided to reach a sufficient denoising result. This is in full accordance with our expectations. When enough steps are provided and no extrapolation is required, both methods are on par with the standard and symmetric ResNets.

A side-by-side comparison yields insights into the performance of different activation functions. We observe that the performance of ReLU networks is only slightly better than classical linear diffusion [57]. This shows that the ReLU is not suited for our denoising problem, regardless of the network architecture. After as few as three network blocks, the improvement of deeper networks is only marginal.

**Fig. 6:**
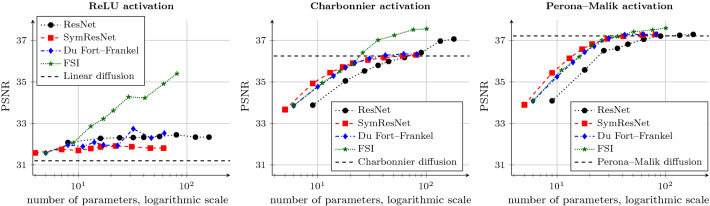
Denoising quality of network architectures with varying depth and a single channel. The parameters are smoothly changing between the residual blocks. Each plot is concerned with a different activation function. The proposed architectures can outperform the standard ResNet by saving a large amount of parameters

In contrast, both the Charbonnier and the Perona–Malik activations are much more suitable. The nonmonotone Perona–Malik activation function yields the best denoising performance, as our diffusion interpretation suggests. When using the original ResNet with a diffusion-inspired activation function, tremendous performance gains in comparison with the ReLU activation can be achieved. This shows that in this experimental setting, the activation is the key to a good performance.

#### Optimality of Diffusion Processes

Interestingly, the standard ResNet with a diffusion activation naturally learns a symmetric filter structure with biases close to 0.

For example, the ResNet with Perona–Malik activation, a grid size of $h=1$, and 20 shared residual blocks learns an inner kernel ${\varvec{k}}_1~=~(0.922, -0.917, 0.006)^\top$, an outer kernel ${\varvec{k}}_2=(0.051, 0.437, -0.489)^\top$ and biases $b_1 = 1.6 \cdot 10^{-1}$ and $b_2 = 1.2 \cdot 10^{-5}$. If we factor out the time step size limit of $\tau =0.5$ for this setting from the outer kernel ${\varvec{k}}_2$, we see that it transforms into ${\varvec{{\tilde{k}}}}_2 = (0.102, 0.874, -0.978)^\top$. It becomes apparent that the kernels approximately fulfil the negated symmetric filter structure.

Moreover, the kernels closely resemble rescaled standard forward and backward difference discretisations. This is surprising, as a kernel of width three allows to learn derivative operators of second order, but a first order operator appears to yield already optimal quality.

This shows that in this constrained setting, second order diffusion processes are an optimal model which is naturally learned by a residual network.

#### Time Dynamic Case

In a practical setting, the residual blocks typically do not share their weights, but train them independently. If the parameters evolve smoothly over the blocks, we can interpret this as an approximation of a time dynamic PDE model.

To investigate the performance of the proposed architectures in this setting, we train the parameters of each block individually, but enforce a certain smoothness between them. If the parameter vector of a block at time level *k* is given by ${\varvec{\theta }}^k$, we add a regulariser

27 $$\begin{aligned} \beta \sum _{k=1}^{K} \tau \left( \frac{{\varvec{\theta }}^{k} - {\varvec{\theta }}^{k-1}}{\tau }\right) ^2 \end{aligned}$$

to the loss function. Here, a smoothness parameter $\beta$ controls the amount of smoothness between the blocks, with higher values of $\beta$ leading to smoother evolutions. This expression approximates the continuous temporal regulariser

28 $$\begin{aligned} \beta \int _{0}^{T} \left( \partial _t {\varvec{\theta }}(t)\right) ^2 \, \mathrm{d}t, \end{aligned}$$

which enforces smoothness of the continuous parameter evolution ${\varvec{\theta }}(t)$. The regularisation ensures that the learned filters change smoothly throughout the layers. This is essential for the numerical scheme to be consistent with the continuous limit case where the step size $\tau$ tends towards 0, see also [93].

For the residual network, where no time step size $\tau$ is learned explicitly, we set the time step size to the inverse of the number of blocks. This requires to use a different smoothness parameter $\beta$. We tune the smoothness parameters for all architectures such that their parameters exhibit similarly smooth evolutions over time. Numerical parameters such as time step size and extrapolation parameters are not affected by the regulariser.

Figure 6 presents the performance of time dynamic architectures with a single channel. We use $\beta =5$ for the standard ResNets, and $\beta =10$ for all other architectures. In contrast to the previous comparisons, we now compare the denoising quality against the number of network parameters. This allows us to measure performance against model complexity.

**Fig. 7:**
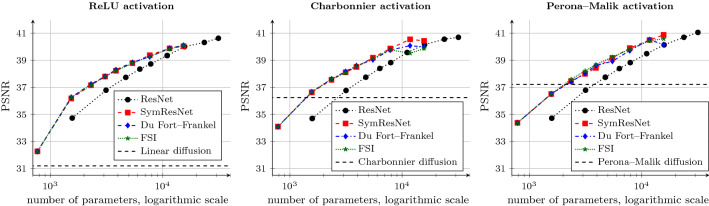
Denoising quality of network architectures with varying depth and $C=16$ network channels. Each plot is concerned with a different activation function. The proposed architectures outperform the standard ResNet for the same amount of parameters. In this setting, the acceleration strategies are on par with the symmetric ResNet, and the margin between activation functions becomes smaller

For the symmetric ResNet, we observe the same behaviour as in the setting without a temporal dynamic. The overall best performance is still on par with the respective classical diffusion process for the Charbonnier and Perona–Malik activation functions.

However, the time dynamic allows this model to achieve better denoising quality for a fewer number of blocks. For example, the symmetric ResNet with Perona–Malik activation and seven residual blocks can achieve a denoising quality of 36.51 dB if weights are shared, but already 37.08 dB with a regularised temporal dynamic. Similar observations for classical diffusion processes with a time dynamic diffusivity function can be found in [36]. Yet, this effectiveness comes at the price of additional parameters.

The Du Fort–Frankel and FSI networks allow for higher efficiency at the cost of more network parameters. Especially in the case of FSI, the trainable extrapolation parameters help to achieve significantly better performance when more residual blocks are provided. This is in accordance with observations in the literature [69].

The proposed architectures outperform the standard ResNet for the same amount of parameters when using Charbonnier and Perona–Malik activations. This shows that the model reduction to a symmetric convolution structure is indeed fruitful. Moreover, the ranking of activation functions remains the same in most cases.

None of the architectures significantly outperform the classical Perona–Malik diffusion process. This supports our claim that these tightly constrained networks realise a numerical algorithm at their core. Different architectures can solve the problem with varying efficiency, but they converge towards the same result. This will only change when we allow for more flexibility within the network architecture, e.g. by utilising multiple network channels.

#### Towards Larger Networks

So far, we have only considered architectures with a single network channel. However, typical CNNs show their full potential when using multiple channels. In this case, the simplicity of the ReLU activation is compensated by a rich set of convolutions between channels.

We now extend our evaluations to architectures with multiple network channels. For simplicity, we leave the number of channels constant throughout the network. To this end, we copy the input signal into *C* channels. The output signal is computed as the average over the individual channel results. In between, we employ the proposed symmetric residual blocks which now use $C\times C$ block convolution matrices with the appropriate stability constraints. These convolutions can be interpreted as ensembles of differential operators which are applied to the signal. The channels are then activated individually and convolved again with the adjoint counterparts of the differential operators.

To allow for maximum flexibility and performance, we remove the temporal parameter regularisation in this experiment.

The denoising performance of the networks is visualised in Fig. 7 for the case of $C=16$ channels.

This experiment is the first instance where all architectures significantly outperform their respective classical diffusion counterparts. The multichannel architecture allows to approximate a more sophisticated denoising model, as information in the various channels is exchanged by means of multichannel convolution operators.

All proposed architectures can still outperform the standard ResNet for the same amount of parameters. In this case, the extrapolation methods are on par with the symmetric ResNet. Interestingly, the ranking of activation functions remains the same, albeit with a much smaller margin. The symmetric ResNet with 20 residual blocks yields PSNR values of 40.04 dB, 40.44 dB and 40.88 dB for the ReLU, Charbonnier and Perona–Malik activations, respectively. We conclude that the more complex the network, the less the activation function matters for performance. On the contrary, this means that networks might be drastically reduced in size when trading network size for sophisticated activation function design.

### Learning a Multigrid Solver for Inpainting

So far, it is not clear if our interpretation of the two-grid FAS is a reasonable model reduction of a full U-net. To prove that this interpretation is indeed of practical relevance, we show how it can be used to learn a multigrid solver for edge-enhancing diffusion inpainting of images [33, 104, 107].

Diffusion-based inpainting aims to restore an image from a sparse set of known data points [33, 107]. Diffusion processes allow for a high reconstruction quality even for extremely sparse known data, making them an interesting tool for image compression applications, see e.g. [33, 98]. Of particular interest is the edge-enhancing diffusion (EED) operator [104] as it allows to reconstruct discontinuous image data such as edges.

As a proof of concept, we construct a network implementing a full multigrid structure. We replace the prescribed nonlinear solvers by trainable feed-forward layers that learn to approximate the PDE at hand.

#### Edge-Enhancing Diffusion Inpainting

The EED inpainting problem can be formulated as follows. Given a set of known image data $f: K\rightarrow {\mathbb {R}}$ on a subset *K* of the image domain $\Omega \subset {\mathbb {R}}^2$, the goal is to compute a reconstruction *u* as the solution of the PDE

29 $$\begin{aligned} (1-c({\varvec{x}})) {\varvec{\nabla }}^\top \!\!\left( {\varvec{D}} {\varvec{\nabla }}u\right) - c({\varvec{x}}) (u-f) = 0. \end{aligned}$$

Here, $c({\varvec{x}})$ is a binary confidence function indicating whether the data at position ${\varvec{x}}$ is known or not. The case $c({\varvec{x}}) = 1$ indicates known data, yielding $u=f$. The case $c({\varvec{x}}) = 0$ indicates that the data need to be reconstructed by EED inpainting. Consequently, $c({\varvec{x}})$ is the characteristic function of the inpainting mask *K*.

The EED operator ${\varvec{\nabla }}^\top \!\!\left( {\varvec{D}} {\varvec{\nabla }}u\right)$ uses a diffusion tensor ${\varvec{D}} = g({\varvec{\nabla }}u_\sigma {\varvec{\nabla }}u_\sigma ^\top )$ based on a Gaussian smoothed gradient ${\varvec{\nabla }}_\sigma$ and a nonlinear diffusivity function *g*. It is a $2 \times 2$ positive semidefinite matrix, which is designed to propagate information along locally dominant structures [104]. As a diffusivity function, we use the Charbonnier diffusivity [18], which relies on a contrast parameter $\lambda$.

#### Experimental Setup

Our trainable FMG architecture is designed as follows: Instead of prescribing nonlinear solvers on each grid, we employ a series of convolutional layers with trainable weights. The remainder of the architecture is fixed: We set the restriction operators to a simple averaging over a $2\times 2$ pixel neighbourhood, and the prolongation operators to nearest neighbour interpolation.

Since FMG employs the same solver on each grid, we realise this idea also in our network by sharing the weights between all solvers for a specific grid. This drastically reduces the amount of parameters and incites that an iterated application of the solvers performs the correct computations.

Instead of training our network by minimising a Euclidean loss between ground truth data and inpainting reconstructions, we use the absolute residual of a discretisation of the inpainting equation (29) as a loss function. This is closely related to the idea of deep energies [37], where one chooses a variational energy as a loss function. Since we do not have such an energy available for EED inpainting, we resort to minimising the absolute residual of the associated Euler–Lagrange equation, which is given by (29). This guarantees that the trained architecture realises EED inpainting as efficiently as possible. To discretise the Euler–Lagrange equation, we employ the standard discretisation from [108].

**Fig. 8:**
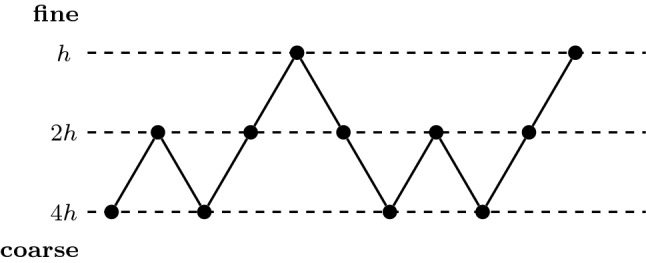
Visualisation of the full multigrid strategy which we employ in our experiments. Dashed horizontal lines denote the three grids, and grids become coarser from top to bottom. Each circle denotes an instance of a solver

**Fig. 9:**
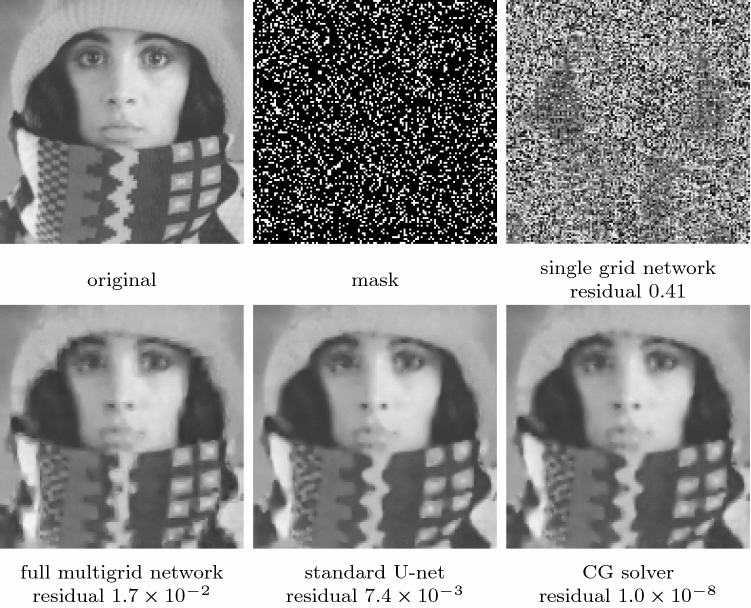
Reconstruction quality of a single grid network, a full multigrid network, a standard U-net, and a classical CG solver for the EED inpainting problem. All results use $\lambda =0.93, \sigma =0.97$ and the same random mask with $20\%$ density. Both the full multigrid network and the standard U-net approximate an EED inpainting result, while a single grid network fails to do so

Whereas classical solution methods for the inpainting problem specify the known data $u=f$ on $\Omega \backslash K$ by means of Dirichlet boundary conditions, we leave it to the network to reproduce also the known data. We have found that this leads to a better approximation quality.

The inpainting masks consist of randomly sampled pixels with a density *d* as a percentage of the number of image pixels. Since the masks are also required on coarser grids to compute the residual within the FMG architecture, we downsample them by putting defining a coarse pixel as known, if at least one pixel in the $2 \times 2$ cell on the fine grid is known.

We train the architecture on a subset of 1000 images of the ImageNet dataset [92] with the Adam optimiser [59] with standard settings.

#### Evaluation of the Full Multigrid Network

We construct a full multigrid network using three grids of size *h*, 2*h* and, 4*h*. The order in which the problem is solved on different grids is given by [4*h*, 2*h*, 4*h*, 2*h*, *h*, 2*h*,  4*h*, 2*h*, 4*h*, 2*h*, *h*]. This is the simplest FMG strategy that can be employed in a setting involving three grids and serves as a proof-of-concept architecture. We visualise this strategy in Fig. 8. Thus, we employ 11 solvers, each using 12 feed-forward convolutional layers with 20 channels and ReLU activations. Weights are shared for solvers on the same grid.

We train this network on an EED inpainting problem with random masks of $20\%$ density. The EED parameters $\lambda =0.93, \sigma =0.97$ have been optimised for inpainting quality with a simple grid search.

To show how the FMG network can benefit from the multigrid structure, we compare it against two networks with the same amount of parameters. One network solves the problem only on a single grid by using 25 layers and 24 channels. Moreover, we compare our FMG network to a standard U-net with addition with three scales, 17 channels and 2 layers per scale. All three models contain $1.2 \times 10^5$ trainable parameters.

Figure 9 shows the inpainting results for the networks at the example of the image *trui*. Moreover, we present the true inpainting result obtained from a conjugate gradient (CG) solver for EED inpainting.

In contrast to the single grid network, the FMG network and the U-net are able to approximate the EED inpainting result. The FMG and U-net results are visually comparable, while the residual of the U-net is slightly better. This does not only show that the multigrid structure is an adequate network design, but also that a standard U-net is not able to obtain a much better solution in this case. From the perspective of numerical algorithms, this is expected, since we know that multigrid methods are highly efficient for these problems.

The advantage of the FMG network is that adding further solvers on the three scales will not inflate the number of parameters as these solvers are shared. This is in contrast to the U-net, where any addition increases the trainable parameter set. The architectural design of the FMG network suggests that U-nets should also be constructed in a similar way. In practice, already concatenating multiple U-nets [75] is a successful idea. Instead of a single down- and upsampling pass, multiple alternating computations on different resolutions should be beneficial.

**Table 1:**
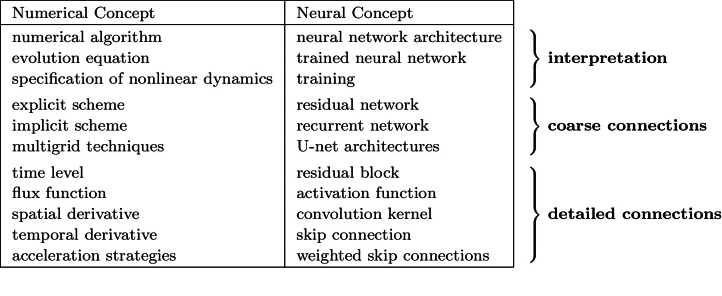
Overview of the connections between numerical and neural concepts which we have encountered

## Discussion and Conclusions

We have shown that numerical algorithms for diffusion evolutions share structural connections to CNN architectures and inspire novel design concepts.

Explicit diffusion schemes yield a specific form of residual networks with a symmetric filter structure, for which one can prove Euclidean stability. Moreover, this architecture saves half of the network parameters, and its stability constraint is easy to implement without affecting its performance. In addition, our connection suggests the use of a diffusion flux function as an activation, revitalising the idea of nonmonotone activation functions. We have shown that these activations perform well for a denoising task, even when using them within standard architectures in a plug-and-play fashion.

By investigating accelerated explicit schemes and implicit schemes, we have justified the effectiveness of skip connections in neural networks. They realise time discretisations in explicit schemes, extrapolation terms to increase their efficiency, and recurrent connections in implicit schemes with fixed point structure. In practice, the resulting architectures are particularly useful when training networks with small amounts of layers.

Lastly, our connection between multigrid concepts and U-net architectures serves as a basis for explaining their success. We have shown that a U-net architecture is able to implement a full multigrid strategy, which allows to learn efficient solutions for PDEs which are typically hard to solve. By directly using the residual norm as a loss function, we can guarantee that the network approximates the PDE at hand. This suggests to extend the standard U-net architecture in a full multigrid fashion.

Our philosophy of identifying numerical concepts as core building blocks of neural architectures has proven to be fruitful. Our direct translation has yielded structural insights into popular neural networks and inspired well-founded neural building blocks with practical relevance. An overview of the detailed connections that we have encountered is presented in Table 1.

Our numerical perspective on neural networks differs from most viewpoints in the literature. However, it provides a blueprint for directly translating a plethora of numerical strategies into well-founded and practically relevant neural building components. We hope that this line of research leads to a closer connection of both worlds and to hybrid methods that unite the stability and efficiency of modern numerical algorithms with the performance of neural networks.

## A Stability Proof for Generalised Du Fort–Frankel Schemes

In this section, we extend the stability proof for linear Du Fort–Frankel Schemes of [41] to the nonlinear setting.

First we rewrite the scheme (20) as a multistep method, obtaining

30 $$\begin{aligned} \begin{pmatrix} {\varvec{u}}^{k+1} \\ {\varvec{u}}^{k} \end{pmatrix} = \begin{pmatrix} \frac{4 \tau \alpha }{1 + 2 \tau \alpha } {\varvec{I}} -\frac{2 \tau }{1 + 2 \tau \alpha } {\varvec{A}}\!\left( {\varvec{u}}^{k}\right) &{} \frac{1 - 2 \tau \alpha }{1 + 2 \tau \alpha } {\varvec{I}} \\ {\varvec{I}} &{} {\varvec{0}} \end{pmatrix} \begin{pmatrix} {\varvec{u}}^{k} \\ {\varvec{u}}^{k-1} \end{pmatrix}.\nonumber \\ \end{aligned}$$

Here, we have abbreviated ${\varvec{A}}({\varvec{u}}^k) = {\varvec{K}}^\top {\varvec{G}}({\varvec{u}}^k) {\varvec{K}}$.

To analyse the stability of the Du Fort–Frankel scheme, we have to show that all eigenvalues of the matrix of the multistep method (30) have an absolute value less than or equal to one. Note that this matrix is not symmetric such that it might have complex eigenvalues.

Let us first define as short-hand notations

31 $$\begin{aligned} \begin{aligned} {\varvec{Q}} {:=}&\ \frac{4 \tau \alpha }{1 + 2 \tau \alpha } {\varvec{I}} - \frac{2 \tau }{1 + 2 \tau \alpha } {\varvec{A}}\!\left( {\varvec{u}}^{k}\right) , \\ {\varvec{B}}{:=}&\ \begin{pmatrix} {\varvec{Q}} &{} \frac{1 - 2 \tau \alpha }{1 + 2 \tau \alpha } {\varvec{I}} \\ {\varvec{I}} &{} {\varvec{0}} \end{pmatrix}. \end{aligned} \end{aligned}$$

We start with the naive approach to compute eigenvalues of ${\varvec{B}}$: $\mu \in {\mathbb {C}}$ is an eigenvalue of ${\varvec{B}}$ if

32 $$\begin{aligned} \det \!\left( {\varvec{B}} - \mu {\varvec{I}}\right) = \det \!\left( \begin{pmatrix} {\varvec{Q}} - \mu {\varvec{I}} &{} \frac{1 - 2 \tau \alpha }{1 + 2 \tau \alpha } {\varvec{I}} \\ {\varvec{I}} &{} - \mu {\varvec{I}} \end{pmatrix} \right) = 0. \end{aligned}$$

As ${\varvec{B}} - \mu {\varvec{I}}$ is a block matrix containing square blocks of the same shape where the lower two blocks commute, we have

33 $$\begin{aligned} \begin{aligned} \det \!\left( {\varvec{B}} - \mu {\varvec{I}}\right)&= \det \!\left( \left( {\varvec{Q}} - \mu {\varvec{I}}\right) (-\mu {\varvec{I}}) - \frac{1-2\tau \alpha }{1+2\tau \alpha } {\varvec{I}} \right) \\&= \det \!\left( \mu ^{2} {\varvec{I}} -\mu {\varvec{Q}} - \frac{1-2\tau \alpha }{1+2\tau \alpha } {\varvec{I}} \right) . \end{aligned} \end{aligned}$$

To proceed from here, it is reasonable to involve the eigenvalues of the matrix ${\varvec{Q}}$. As ${\varvec{Q}}$ is real-valued and symmetric, there exist an orthogonal matrix ${\varvec{V}}$ of eigenvectors of ${\varvec{Q}}$ and a diagonal matrix ${\varvec{\varGamma }}$ with the eigenvalues $\gamma$ of ${\varvec{Q}}$ on its diagonal such that

34 $$\begin{aligned} {\varvec{Q}} = {\varvec{V}} {\varvec{\varGamma }} {\varvec{V}}^{T}. \end{aligned}$$

As ${\varvec{V}}$ is an orthogonal matrix, it holds that

35 $$\begin{aligned} {\varvec{I}} = {\varvec{V}} {\varvec{V}}^{T}. \end{aligned}$$

Plugging both of these relations into (32) yields

36 $$\begin{aligned}&\det \!\left( {\varvec{B}} - \mu {\varvec{I}}\right) \nonumber \\&\quad = \det \!\left( \mu ^{2} {\varvec{V}} {\varvec{V}}^{T} -\mu {\varvec{V}} {\varvec{\varGamma }} {\varvec{V}}^{T} - \frac{1-2\tau \alpha }{1+2\tau \alpha } {\varvec{V}} {\varvec{V}}^{T} \right) \nonumber \\&\quad = \det \!\left( {\varvec{V}} \left( \mu ^{2} {\varvec{I}} -\mu {\varvec{\varGamma }} - \frac{1-2\tau \alpha }{1+2\tau \alpha } {\varvec{I}} \right) {\varvec{V}}^{T} \right) \nonumber \\&\quad =\det \!\left( {\varvec{V}}\right) \det \!\left( \mu ^{2} {\varvec{I}} -\mu {\varvec{\varGamma }} - \frac{1-2\tau \alpha }{1+2\tau \alpha } {\varvec{I}} \right) \det \!\left( {\varvec{V}}^{T}\right) \nonumber \\&\quad = \det \!\left( \mu ^{2} {\varvec{I}} -\mu {\varvec{\varGamma }} - \frac{1-2\tau \alpha }{1+2\tau \alpha } {\varvec{I}} \right) , \end{aligned}$$

since the determinants of the orthogonal matrices ${\varvec{V}}$ and ${\varvec{V}}^{T}$ are both 1. The remaining determinant is concerned with a diagonal matrix. Thus, it is equal to the product of the diagonal elements, i.e.

37 $$\begin{aligned} \det \!\left( {\varvec{B}} - \mu {\varvec{I}}\right) = \prod _{j=1}^{N} \left( \mu ^{2} -\mu \, \gamma _{j} - \frac{1-2\tau \alpha }{1+2\tau \alpha } \right) . \end{aligned}$$

For $\mu$ to be an eigenvalue of ${\varvec{B}}$, we need that this product vanishes. This is exactly the case if one or more factors in the product vanish. Hence, we get that for a fixed eigenvalue $\gamma$ of ${\varvec{Q}}$, an eigenvalue $\mu$ of ${\varvec{B}}$ satisfies

38 $$\begin{aligned} \mu = \frac{\gamma }{2} \pm \sqrt{\frac{\gamma ^{2}}{4} + \frac{1-2\tau \alpha }{1+2\tau \alpha }}. \end{aligned}$$

For stability of (30), we need that $|\mu | \le 1$ for all solutions $\mu$ for all eigenvalues $\gamma$ of ${\varvec{Q}}$. To proceed further, we consider the discriminant $\frac{\gamma ^{2}}{4} + \frac{1-2\tau \alpha }{1+2\tau \alpha }$. Since we know that ${\varvec{Q}}$ is real-valued and symmetric, it follows that $\gamma$ is a real number. Thus, $\gamma ^{2}$ is positive. We also know that $\tau$ and $\alpha$ are positive, such that

39 $$\begin{aligned} -1< \frac{1-2\tau \alpha }{1+2\tau \alpha } < 1. \end{aligned}$$

Therefore, it is possible for the discriminant to have negative values, which results in complex eigenvalues $\mu$. Let us therefore distinguish the three cases of negative discriminant, vanishing discriminant, and positive discriminant. We will see that the last one is the only case which introduces a lower bound on $\alpha$ for unconditional stability.

*Vanishing Discriminant* First, we consider a vanishing discriminant, which can only happen if $2\tau \alpha \ge 1$. This yields

40 $$\begin{aligned} \gamma = \pm \, 2 \,\sqrt{\frac{2\tau \alpha -1}{2\tau \alpha +1}}. \end{aligned}$$

Thus, the eigenvalues of ${\varvec{B}}$ are given by

41 $$\begin{aligned} \mu = \frac{\gamma }{2} = \pm \sqrt{\frac{2\tau \alpha -1}{2\tau \alpha +1}}. \end{aligned}$$

Since the fraction takes values between 0 and 1, the same is true for the square root. Therefore, we have $|\mu | < 1$.

*Negative Discriminant* If the discriminant is negative, the corresponding values of $\mu$ are complex and we can write

42 $$\begin{aligned} \mu = \frac{\gamma }{2} \pm \mathrm {i} \, \sqrt{-\frac{\gamma ^{2}}{4} - \frac{1-2\tau \alpha }{1+2\tau \alpha }}. \end{aligned}$$

Then we get for the squared absolute value of $\mu$

43 $$\begin{aligned} \left|\mu \right|^{2} = \frac{\gamma ^2}{4} + \left( -\frac{\gamma ^{2}}{4} - \frac{1-2\tau \alpha }{1+2\tau \alpha }\right) = \frac{2\tau \alpha -1}{2\tau \alpha +1}. \end{aligned}$$

Surprisingly, this does not depend on $\gamma$ and we recover once more the condition

44 $$\begin{aligned} -1< \frac{2\tau \alpha -1}{2\tau \alpha +1} < 1. \end{aligned}$$

Thus, we again have $|\mu | < 1$.

*Positive Discriminant* In this case, the eigenvalues $\mu$ are real-valued. Thus, we get the condition

45 $$\begin{aligned} -1< \frac{\gamma }{2} \pm \sqrt{\frac{\gamma ^{2}}{4} + \frac{1-2\tau \alpha }{1+2\tau \alpha }} < 1. \end{aligned}$$

If $\tau > 0$ and $\alpha > 0$, this system has the following solutions for $\gamma$:

46 $$\begin{aligned} \begin{aligned}&|\gamma |< \frac{4\tau \alpha }{1+2\tau \alpha },&\text {if } \; 0 \le \frac{1-2\tau \alpha }{1+2\tau \alpha }< 1, \\ \sqrt{\frac{8\tau \alpha -4}{2\tau \alpha +1}} \le \,&|\gamma |< \frac{4\tau \alpha }{1+2\tau \alpha },&\text {if } -1< \frac{1-2\tau \alpha }{1+2\tau \alpha } < 0. \end{aligned}\nonumber \\ \end{aligned}$$

If we treat $\gamma$ as a complex number for the moment, the first condition means that $\gamma$ has to be inside a disc of radius $\frac{4\tau \alpha }{1+2\tau \alpha }$ around the origin and the second condition means that $\gamma$ has to be inside that disc, but outside or on the boundary of a second disk of radius $\sqrt{\frac{8\tau \alpha -4}{2\tau \alpha +1}}$. Hence, the second condition is more restrictive.

It remains to determine conditions on $\tau$ and $\alpha$ such that (46) is always satisfied for all eigenvalues $\gamma$ of the matrix ${\varvec{Q}}$. As the matrix ${\varvec{A}}({\varvec{u}}^k)$ is real-valued and symmetric, we can diagonalise it in the same fashion as ${\varvec{Q}}$: There exist an orthogonal matrix ${\varvec{W}}$ and a diagonal matrix ${\varvec{\Lambda }}$ with the eigenvalues $\lambda$ on its diagonal such that

47 $$\begin{aligned} {\varvec{A}}({\varvec{u}}^k) = {\varvec{W}} {\varvec{\Lambda }}{\varvec{W}}^\top . \end{aligned}$$

With the help of this representation, we can write

48 $$\begin{aligned} \begin{aligned} {\varvec{Q}}&= \frac{4 \tau \alpha }{1 + 2 \tau \alpha } {\varvec{I}} - \frac{2 \tau }{1 + 2 \tau \alpha } {\varvec{A}}\!\left( {\varvec{u}}^{k}\right) \\&= \frac{4 \tau \alpha }{1 + 2 \tau \alpha } {\varvec{W}} {\varvec{W}}^{T} - \frac{2 \tau }{1 + 2 \tau \alpha } {\varvec{W}} {\varvec{\Lambda }} {\varvec{W}}^{T} \\&={\varvec{W}} \left( \frac{4 \tau \alpha }{1 + 2 \tau \alpha } {\varvec{I}} - \frac{2 \tau }{1 + 2 \tau \alpha } {\varvec{\Lambda }} \right) {\varvec{W}}^{T}. \end{aligned} \end{aligned}$$

Hence, the eigenvalues of ${\varvec{Q}}$ are given by

49 $$\begin{aligned} \gamma = \frac{4 \tau \alpha }{1 + 2 \tau \alpha } - \frac{2 \tau }{1 + 2 \tau \alpha } \lambda , \end{aligned}$$

where $\lambda$ is an eigenvalue of ${\varvec{A}}({\varvec{u}}^{k})$.

With this formula, the first case of (46) reduces to

50 $$\begin{aligned} 0< \lambda < 4 \alpha . \end{aligned}$$

This condition is similar to the stability condition of the explicit scheme, however now for $\alpha$ instead of $\frac{1}{\tau }$. The estimate on the left-hand side is always fulfilled if ${\varvec{A}}({\varvec{u}}^k)$ is positive definite. For now, we assume that ${\varvec{A}}({\varvec{u}}^k)$ is positive definite and consider the case of a zero eigenvalue afterwards.

The inequality (50) has to hold for every eigenvalue of ${\varvec{A}}({\varvec{u}}^k)$. If ${\varvec{A}}({\varvec{u}}^k)$ is positive definite, we can replace $\lambda$ by the spectral radius $\rho ({\varvec{A}}({\varvec{u}}^k))$ as an upper bound. Hence, we arrive at

51 $$\begin{aligned} \alpha > \frac{\rho \!\left( {\varvec{A}}({\varvec{u}}^k)\right) }{4}. \end{aligned}$$

This is in line with the result that has been derived for the linear case [41].

For the second case in (46), plugging in (49) and simplifying yields

52 $$\begin{aligned} \begin{aligned}&0< \lambda \le 2 \alpha - \sqrt{4\alpha ^{2}-\frac{1}{\tau ^{2}}} \qquad \text {or} \\&2 \alpha + \sqrt{4\alpha ^{2}-\frac{1}{\tau ^{2}}} \le \lambda < 4\alpha \end{aligned} \end{aligned}$$

As we are in the case in which $-1< \frac{1-2\tau \alpha }{1+2\tau \alpha } < 0$, the square root is a nonnegative real number.

Moreover, we have to investigate what happens if

53 $$\begin{aligned} \begin{aligned}&-1 \le \frac{1-2\tau \alpha }{1+2\tau \alpha }< 0 \qquad \text {and} \\&2 \alpha - \frac{\sqrt{4\alpha ^{2}\tau ^{2}-1}}{\tau ^{2}}< \lambda < 2 \alpha + \frac{\sqrt{4\alpha ^{2}\tau ^{2}-1}}{\tau ^{2}}. \end{aligned} \end{aligned}$$

With some tedious but straight-forward computations, one can show that this case corresponds exactly to the case with a negative discriminant, which did not introduce any new stability conditions.

Lastly, we consider the case where ${\varvec{A}}({\varvec{u}}^k)$ is only positive semidefinite. If $2\tau \alpha < 1$, then $\lambda =0$ lies in the range given for $\lambda$ in (53). For this case, we have already shown that $|\mu |<1$ and the scheme is stable.

If $2\tau \alpha >1$, we obtain from (49):

54 $$\begin{aligned} \gamma = \frac{4 \tau \alpha }{1 + 2 \tau \alpha }. \end{aligned}$$

We can plug this value of $\gamma$ into (40) to see that $\mu =1$ is always a solution in this case. We have to ensure that all other solutions for $\mu$ fulfil $|\mu |<1$. The other solution of (40) for $\lambda =0$ is

55 $$\begin{aligned} \mu = \frac{2 \tau \alpha - 1}{2 \tau \alpha + 1}, \end{aligned}$$

which yields $|\mu | < 1$ since $\tau > 0$ and $\alpha > 0$. All other eigenvalues of ${\varvec{B}}$ have an absolute value of less than one by the considerations above. Thus, we can conclude that also in this case, the Du Fort–Frankel scheme is stable.

Consequently, the only stability condition on $\alpha$ is the one in (51). In a similar manner as for the symmetric ResNet, we can transform this condition into an a priori constraint

56 $$\begin{aligned} \alpha \ge \frac{L}{4} . \end{aligned}$$
